# Exact Analytical Model for Bose-Einstein Condensate at Negative Temperature

**DOI:** 10.1038/s41598-020-65765-9

**Published:** 2020-06-02

**Authors:** Ajay Nath, Jayanta Bera, Suranjana Ghosh, Utpal Roy

**Affiliations:** 1 Indian Institute of Information Technology Vadodara, Gandhinagar, 382028 India; 2 Department of Physics, Indian Institute of Technology Patna, Bihta, Patna, 801106 India; 3 Indian Institute of Science Education and Research Kolkata, Mohanpur, 741246 India

**Keywords:** Ultracold gases, Matter waves and particle beams

## Abstract

We present an exact analytical model of a cigar-shaped Bose-Einstein condensate at negative temperature. This work is motivated by the first experimental discovery of negative temperature in Bose-Einstein condensate by Braun *et al*. We have considered an external confinement which is a combination of expulsive trap, bi-chromatic optical lattice trap, and linear trap. The present method is capable of providing the exact form of the condensate wavefunction, phase, nonlinearity and gain/loss. One of the consistency conditions is shown to map onto the Schrödinger equation, leading to a significant control over the dynamics of the system. We have modified the model by replacing the optical lattice trap by a bi-chromatic optical lattice trap, which imparts better localization at the central frustrated site, delineated through the variation of condensate fraction. Estimation of temperature and a numerical stability analysis are also carried out. Incorporation of an additional linear trap introduces asymmetry and the corresponding temporal dynamics reveal atom distillation at negative temperature.

## Introduction

Negative temperature is the state of a system in which slope of entropy *w.r.t*. internal energy of the system is negative^1–3^. At negative temperature, occupancy of atoms is greater at higher energy levels. The idea of negative absolute temperature is not new^4^. In 1951, Purcell and Pound introduced the concept of negative temperature in the context of spin system^5,6^. A useful introduction to negative temperature in thermodynamics is also furnished by Landau and Lifshitz^7^ and by Kittel and Kroemer^8^. The reason why negative temperature is rarely observed in reality is that it is very non-trivial to realize an upper bound of energy in equilibrium. Usually, in most of the systems, the energy is lower bound and only positive absolute temperature, i.e. *T* > 0, is allowed in equilibrium^1^. The experimental conditions required for the existence of a stable negative temperature state with bosons are attractive interactions and an anti-trapping potential. As a result, all three kinds of energy: kinetic energy, potential energy and interaction energy, have their upper bound and atoms can pile up in the higher energy state at equilibrium. Recently, Braun *et al*. in a pioneering experiment realized negative temperature for the first time in a physical system of Bose-Einstein condensate (BEC)^9^. In the experimental arrangement, a transition from harmonic to expulsive trap, superimposed over an optical lattice, is performed for achieving higher occupation of atoms at higher energy states^9–12^. In recent times, negative temperature has been investigated for spin vortices^13^, cosmology^14^, quantum fluctuations^15^, definition of entropy^16–18^, photo-induced state^19^, work storage in states^20^ and votices from chaos^21^ *etc*.

In this paper, we provide an exact analytical model for the dynamics of cigar-shaped condensate at negative temperature. We have considered the external confinement, primarily, a combination of an expulsive and a bi-chromatic optical lattice (BOL) potentials. The motivation for considering BOL trap are threefold: (i) conversion to optical lattice (OL): BOL is generated by the superposition of two OL’s of different wavelengths and intensities^22^. By tuning the power and the wavelength of the constituent laser beams, one can create a pure OL in a special case and vice-versa, allowing precise control over the shape of the trap profile; (ii) simulator for other systems like condensed matter physics in the context of supersolidity^23–25^; (iii) rich in physical phenomena: a number of interesting phenomena are already observed in BOL making it a suitable test-bed for studying them in negative temperature scenario^26–31^. In addition to BOL, we have also added a linear trap for incorporating an overall asymmetry to the potential. Utilizing this asymmetry, a novel mechanism for atom distillation is demonstrated, which can have novel applications towards quantum information processing at negative temperature. We calculate the exact form of the wavefunction, phase, nonlinearity and gain/loss. Further, we find that one of the consistency conditions governing the condensate dynamics interestingly maps the Schrödinger equation. This leads to a significant control over the dynamics of the system. Although from this novel procedure, a family of solutions are obtained, we mainly emphasize on the localized non-linear solitary excitations^32^. We investigate the variation of atom number density and filling fraction with the depth of the BOL and strength of the expulsive oscillator. The localization of condensate density, which is the characteristic feature of a disordered potential, is observed corresponding to the variation of trap parameters in the negative temperature domain. We also identify that the oscillator frequency and the intensity of the laser beams act as the key trap parameter for controlling the rate of localization in the system.

In the following section, we present the model for constructing exact solution of 1D-Gross Pitäevskii equation (GPE) at negative temperature under the composite confinement (a linear trap, an expulsive oscillator and a BOL trap) in presence of space- and time-modulated cubic nonlinearity and gain/loss. Here, we have considered (not limited to) the system with weak and attractive interatomic interaction. The methodology for calculating the system variables is explicated by finding the travelling coordinate, wavefunction, nonlinearity, gain/loss and various trap parameters. It is shown that by tuning the oscillator frequency one can generate various potentials like a mixture of harmonic-BOL, and a mixture of expulsive-BOL. Moreover, one of the consistency conditions governing the dynamics of condensate is connected to the linear Schrödinger eigenvalue problem, which allows one to obtain a wide variety of temporal variations for each solvable quantum mechanical potential. Although, we have mainly emphasized on the localized excitations for instance, a family of solutions (both localized and periodic) can be obtained from our method. We then investigate the dynamics of condensate under the influence of above-mentioned confinement. The axial compression of the atomic number density is observed with the variation of trap parameters, *i.e*., the power and wavelength of the lasers, and the expulsive oscillator frequency. For a better insight of the phenomenon of localization, the profile of occupation density is also plotted. The key tuning parameters responsible for axial compression are identified. A confirmation of negative temperature is presented through an estimation of temperature from formal definition and a numerical stability analysis. Moreover, by tuning the asymmetry component of the trap, we achieve transport of condensate atoms across the potential barriers.

## Results

### Analytical model

We start by writing a general form of the dimensionless 1D GPE:

1 $$i\frac{\partial \psi (z,t)}{\partial t}=-\frac{1}{2}\frac{{\partial }^{2}\psi (z,t)}{\partial {z}^{2}}+g(z,t)|\psi (z,t{)|}^{2}\psi (z,t)+V(z,t)\psi (z,t)+i\tau (z,t)\psi (z,t),$$

where *ψ*(*z*, *t*) is the wavefunction of the condensate and *g*(*z*, *t*) is the nonlinearity, arising due to atom-atom interaction. *τ*(*z*, *t*), being the coefficient of imaginary term, represents loss or gain of condensate atoms^33,34^. The coefficients of the starting Eq. (1) are taken space- and time-dependent for making the present model more generous. The external trap, *V*(*z*, *t*), is of the form

2 $$V(z,t)=M(t){z}^{2}+{V}_{1}(t)\cos \,\mathrm{[2}l\{N(t)z+O(t)\}]+{V}_{2}(t)\cos \,[l\{N(t)z+O(t)\}]+P(t)z\mathrm{}.$$

Frequencies of the two laser beams are commensurate and *V*_1_(*t*), *V*_2_(*t*) are their potential depths. The potential depths are expressed in terms of the recoil energy and can be controlled through the wavelength of the laser (*λ*) and the mass of the atoms (*m*_*c*_). *l* = 2*πa*_⊥_/*λ* is the lattice wave vector for transverse oscillator length ${a}_{\perp }=\sqrt{\hslash /{m}_{c}{\omega }_{\perp }}$. *N*(*t*), *O*(*t*) are time-dependent parameters and *P*(*t*) controls the amount of asymmetry in the resulting potential. *ω*_⊥_ is the transverse frequency of the cigar shaped trap. In order to solve Eq. (1), we take a general form of the ansatz:

3 $$\psi (z,t)=A(z,t)F[Z(z,t)]{e}^{i\theta (z,t)}\mathrm{}.$$

*A*(*z*, *t*) and *θ*(*z*, *t*) are the amplitude and phase of the condensate, respectively. *F*[*Z*(*z*, *t*)] is a real function, which will manifest condensate density. The above ansatz leads to the following consistency conditions:

4 $$\begin{array}{c}\frac{\partial A}{\partial z}\frac{\partial Z}{\partial z}+\frac{1}{2}A\frac{{\partial }^{2}Z}{\partial {z}^{2}}=0,\,\frac{\partial Z}{\partial t}+\frac{\partial Z}{\partial z}\frac{\partial \theta }{\partial z}=0,\\ G{\left(\frac{\partial Z}{\partial z}\right)}^{2}-2{A}^{2}g=\mathrm{0,}\,\frac{\partial A}{\partial t}+\frac{\partial A}{\partial z}\frac{\partial \theta }{\partial z}+\frac{A}{2}\frac{{\partial }^{2}\theta }{\partial {z}^{2}}-\tau A=\mathrm{0,}\\ V=\frac{1}{2A}\frac{{\partial }^{2}A}{\partial {z}^{2}}-\frac{1}{2}{\left(\frac{\partial \theta }{\partial z}\right)}^{2}-\frac{\partial \theta }{\partial t},\,\frac{{\partial }^{2}F}{\partial {Z}^{2}}-G{F}^{3}=0.\end{array}$$

For convenience, we have written: *A* ≡ *A*(*z*, *t*), *V* ≡ *V*(*z*, *t*), *F* ≡ *F*[*Z*(*z*, *t*)], *θ* ≡ *θ*(*z*, *t*), *g* ≡ *g*(*z*,*t*), *τ* ≡ *τ*(*z*, *t*) and *Z* ≡ *Z*(*z*, *t*). *G* is a real constant related to the nolinearity coefficient. The last consistency condition in Eq. (4) is nothing but the elliptic equation, whose solutions are known in the form of 12 Jacobian elliptic functions. The solutions vary from periodic to localized for different values of the modulus parameter (0 < *m* < 1)^35^. Simultaneous solution of all the above consistency conditions is tricky and needs a proper sequence of analytical step to end up with exact expressions of the equation parameters. Equation (4) thus reduces to

5 $$\begin{array}{rcl}A & = & \sqrt{c(t){\left(\frac{\partial Z}{\partial z}\right)}^{-1}},\,\theta =-\,\int \frac{\partial Z}{\partial t}{\left(\frac{\partial Z}{\partial z}\right)}^{-1}dz,\,g=\frac{G}{2c(t)}{\left(\frac{\partial Z}{\partial z}\right)}^{3},\\ \tau  & = & \left[\frac{1}{2c(t)}\frac{\partial c(t)}{\partial t}{\left(\frac{\partial Z}{\partial z}\right)}^{2}-\frac{\partial Z}{\partial z}\frac{\partial }{\partial t}\left(\frac{\partial Z}{\partial z}\right)+\frac{{\partial }^{2}Z}{\partial {z}^{2}}\frac{\partial Z}{\partial t}\right]\times {\left(\frac{\partial Z}{\partial z}\right)}^{-2}\mathrm{}.\end{array}$$

*c*(*t*) is a positive definite function of time. The exact form of the traveling coordinate *Z* is subsequently evaluated:

6 $$Z={\int }_{0}^{\xi }{e}^{\beta \cos (l\xi {\prime} )}d\xi {\prime} ,$$

where, *Z* = *f*[*ξ*(*z*, *t*)] with *ξ*(*z*, *t*) = *γ*(*t*)*z* + *ζ*(*t*). *γ*(*t*) and *ζ*(*t*) are functions of time and the other parameters are connected in the following manners.

7 $$\begin{array}{c}{V}_{1}(t)=-\,\frac{{\beta }^{2}{l}^{2}{\gamma }^{2}(t)}{16},\,{V}_{2}(t)=\frac{\beta {l}^{2}{\gamma }^{2}(t)}{4},\\ N(t)=\gamma (t),\,O(t)=\zeta (t),M(t)={(2{\gamma }^{2}(t))}^{-1}[\gamma {\prime\prime} (t)\gamma (t)-2\gamma {{\prime} }^{2}(t)],\\ P(t)={({\gamma }^{2}(t))}^{-1}[\zeta {\prime\prime} (t)\gamma (t)-2\zeta (t)\gamma {\prime} (t)],\\ \alpha (t)={\int }_{0}^{t}\left[\frac{{\beta }^{2}{l}^{2}{\gamma }^{2}(t)}{16}-\frac{{\zeta {\prime} }^{2}(t)}{2{\gamma }^{2}(t)}\right]dt{\prime} \mathrm{}.\end{array}$$

Here, the constant parameters, *l* and *β*, help to control the central frequency and the intensity of the laser beams, whereas *α*(*t*) is constant of integration arising from the equation involving the phase. Finally, the expressions of the physical parameters become

8 $$\begin{array}{c}A=\sqrt{\frac{c(t)}{{e}^{\beta cos[l(\gamma (t)z+\zeta (t))]}}},\,\theta =-\,\frac{\gamma {\prime} (t)}{2\gamma (t)}{z}^{2}-\frac{\zeta {\prime} (t)}{\gamma (t)}z+\alpha (t),\\ g=\frac{G{\gamma }^{3}(t)}{2c(t)}{e}^{3\beta cos[l(\gamma (t)z+\zeta (t))]},\,\tau =\left[\frac{1}{2}\frac{c{\prime} (t)}{c(t)}-\frac{\gamma {\prime} (t)}{\gamma (t)}\right],\end{array}$$

where *M*(*t*) is the oscillator frequency of the quadratic component of the external trap. It is worth mentioning that a substitution, $\gamma (t)=\frac{1}{\nu (t)}$, in the expression of *M*(*t*) in Eq. (7) leads to

9 $$\nu {\prime\prime} (t)+2\nu (t)M(t)=0,$$

which is already in the form of the well-known Schrödinger equation. Further, another transformation, $\nu (t)={e}^{-{\int }_{0}^{t}\kappa (t{\prime} )dt{\prime} }$, allows Eq. (9) to write in the form of Riccati equation,

10 $$\kappa {\prime} (t)-{\kappa }^{2}(t)=2M(t).$$

By utilizing the merit of these connections, corresponding to each solvable quantum-mechanical system, one can identify the dynamics of the solitonic excitation. The fact that the Schrödinger equation and the Riccati equation can be exactly solved for a variety of *M*(*t*) gives us freedom to control the dynamics of the BEC in a number of analytically tractable ways. Thus, the complete solution of Eq. (1) can be written as

11 $$\psi (z,t)=\sqrt{\frac{c(t)}{\gamma (t){e}^{\beta \cos (l\xi )}}}cn[{\int }_{0}^{\xi }{e}^{\beta \cos (l\xi {\prime} )}d\xi {\prime} ,m]{e}^{i\theta (z,t)}\mathrm{}.$$

In general, Eq. (11) signifies a family of exact solution, depending upon the choice of the modulus parameter of the Jacobi elliptic function (*m*). However, we emphasize only on the localized excitation for specific illustration of our result.

### More insight of the trap profile

By choosing the suitable forms of *γ*(*t*) and *ζ*(*t*), one can create various types of external confinement. However, in order to focus on the main goal of this work, *i*.*e*., to achieve the negative temperature scenario, which requires bound states in higher energy level, we consider the form of the potential as

12 $$V(z,t)=M{z}^{2}+{V}_{1}(t){\rm{\cos }}\mathrm{[2}l\{\gamma (t)z+\zeta (t)\}]+{V}_{2}(t){\rm{\cos }}[l\{\gamma (t)z+\zeta (t)\}]+P(t)z\mathrm{}.$$

The first term in the potential is the harmonic trap which can be made confining or expulsive depending on the sign of *M*. In the present case, *M* is taken as negative to make it expulsive. Second and third terms constitute to form a BOL, which can temporally be shaken by modulating *ζ*(*t*). The fourth term is a spatially linear term, which is added to incorporate asymmetry in the potential. Here, we consider $\gamma (t)=\gamma \text{sec}[\sqrt{2Mt}]$ and $\zeta (t)=p\,\cos (t)\gamma (t)/(1-2M)$, with *γ*, *M* and *p* being real constants. The other potential parameters can be expressed as

13 $${V}_{1}(t)=-\frac{{\beta }^{2}{l}^{2}{\gamma }^{2}{\sec }^{2}[\sqrt{2Mt}]}{16},\,{V}_{2}(t)=\frac{\beta {l}^{2}{\gamma }^{2}{\sec }^{2}[\sqrt{2Mt}]}{4},\,P(t)=p\,\cos (t\mathrm{)}.$$

The above potential can produce the following physical situations:

(i) *ζ*(*t*) = 0, *M* = 0; the potential is a pure BOL,
(ii) *ζ*(*t*) = 0, *M* < 0; the potential is a mixture of expulsive harmonic and BOL,
(iii) *ζ*(*t*) = 0, *M* > 0; the potential is a mixture of confining harmonic and BOL,
(iv) *ζ*(*t*) > 0 or *ζ*(*t*) < 0; the potential is asymmetric.

**Figure 1:**
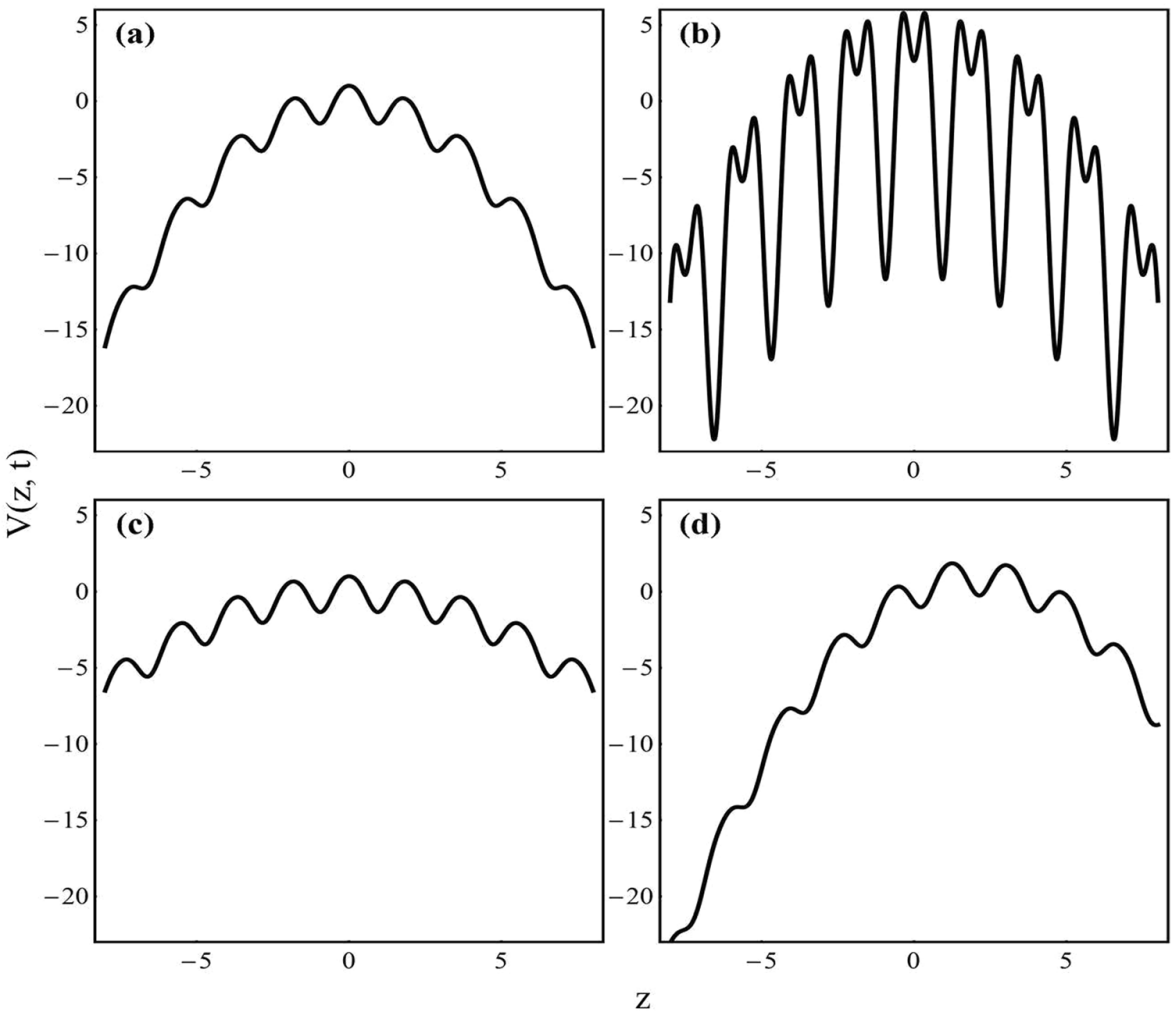
Variation of the external confinement: (**a**) combination of expulsive and OL for *β* = 0.4, *M* = −0.25 and *p* = 0; (**b**) combination of expulsive and BOL for *β* = 2.5, *M* = −0.25, and *p* = 0; (**c**) combination of expulsive and OL with different *M* for *β* = 0.4, *M* = −0.1, *p* = 0; and (**d**) combination of linear, expulsive and BOL for *β* = 0.4, *M* = −0.25, *p* = 1. Other parameters are *γ* = 4, *l* = 0.84 and *t* = 0. Position coordinate is scaled by the harmonic oscillator length.

It is evident from Eq. (12) that by modulating *β*, *M* and *p*, one can tune the potential depths, period and asymmetry of the composite trap. As an illustration, in Fig. (1), we depict the effective trap profile for (a) expulsive-OL: *β* = 0.4, *M* = −0.25, *p* = 0; (b) expulsive-BOL: *β* = 2.5, *M* = −0.25, *p* = 0; (c) expulsive-OL for different value of *M*: *β* = 0.4, *M* = −0.1, *p* = 0; and (d) linear-expulsive-OL: *β* = 0.4, *M* = −0.25, *p* = 1. The other parameters are taken as *γ* = 4, *l* = 0.84 and *t* = 0. In Fig. 1(a), an expulsive-OL configuration is depicted, which is the type of confinement considered in the first experimental realization of negative temperature in BEC^9^. The effect of the power of the laser beam (*β*) is depicted in Fig. 1(b), where *β* increases from 0.4 → 2.5, resulting into the formation of expulsive-BOL. In Fig. 1(c), the role of the strength of oscillator frequency (*M* changes from −0.25 to −0.1) is illustrated in comparison to Fig. 1(a). The variation of the stiffness of the expulsive trap is also apparent from Eq. (12). Finally, in Fig. 1(d), the trap configuration is depicted in presence of an asymmetry. Here, *p* varies from 0 to 1. Depending on the need of the physical situation, one can choose the parameters corresponding to the depth, period, width and asymmetry of the trap.

### Dynamics of condensate density at negative temperature

In this section, we will investigate the dynamics of cigar-shaped condensate in a negative temperature framework. Here, we have taken *G* = −1, i.e. attractive interatomic interaction and for simplicity, *c*(*t*) = *γ*^2^(*t*), i.e. zero gain/loss in the system. Thus, the modified form of the wavefunction becomes:

14 $$\psi (z,t)=\sqrt{\frac{\gamma (t)}{{e}^{\beta \cos (l\xi )}}}cn[{\int }_{0}^{\xi }{e}^{\beta \cos (l\xi {\prime} )}d\xi {\prime} ,m]{e}^{i\theta (z,t)}\mathrm{}.$$

where $$\xi (z,t)=\gamma \text{sec}[\sqrt{2Mt}]z+p\,\cos (t)\gamma (t)/(1-2M)$$.

**Figure 2:**
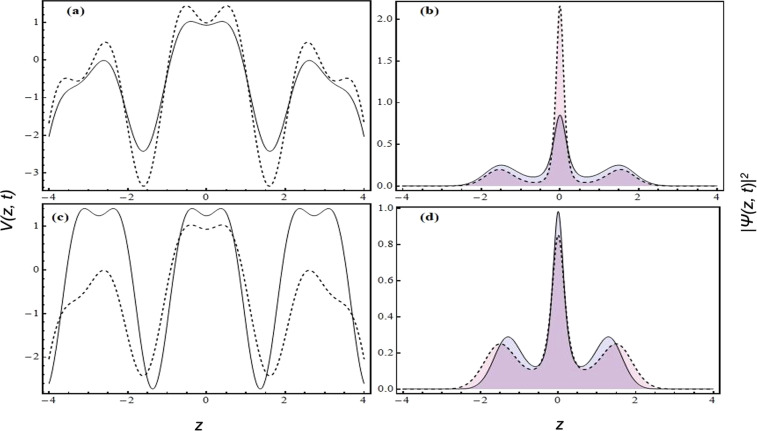
The condensate densities are displayed along with the corresponding trap profiles for varying *β* and *M*. (**a**) Expulsive-BOL confinement for *β* = 1.5 (solid line) and *β* = 2 (dashed line) with *M* = −0.15; (**b**) Condensate density for the same values of *β* and *M* as (**a,c**) Expulsive-BOL confinement for *M* = 0 (dashed line) and *M* = −0.15 (solid line) with *β* = 1.5; (**d**) Condensate density for the same values of *M* and *β* as (**c**). Other parameters are *γ* = 1.25, *l* = 1.84, *p* = 0, *G* = −1, *m* = 1 and *t* = 1. All parameters are in dimensionless unit.

In order to investigate the dynamics of condensate density at negative temperature for expulsive-BOL traps, we ponder upon the specific values of the trap parameters: *M* < 0 and *p* = 0, where *γ* = 1.25, *G* = −1, *t* = 1, *l* = 1.84 and *m* = 1. Figure 2(a) depicts the variation of expulsive-BOL with the laser intensity *β*. It can be inferred from Fig. 2(a) that with increasing the magnitude of *β* (1.5 → 2), the BOL term of the trap becomes dominant over the expulsive term and correspondingly the depths of BOL lattices increase. Here, we have taken *β* = 1.5 (solid line) and *β* = 2 (dashed line) with *M* = −0.15. Figure 2(b) illustrates the variation of condensate density (depicted by the filled plots) with the laser intensity for the same parameters of Fig. 2(a). The solid and dotted lines in the density plot correspond to the same parameters with the potential plots. It clearly exhibits the axially compression of number density with *β*. This axial compression of atom number density can be attributed to the depth of lattice site and also the depth of lattice frustration^30,31^, gradually showing the localization of condensate atoms towards the central lattice site due to the presence of disorder potential^28^. This localization of atom density indicates the increase in negative temperature. Further, in Fig. 2(c), we consider another scenario, where the role of the stiffness of the expulsive term is revealed. Here, the confinement profile is depicted for *M* = 0 (solid line) and *M* = −0.15 (dashed line) with *β* = 1.5, *γ* = 1.25, *l* = 0.84, *p* = 0, and *t* = 1. Corresponding condensate density at different lattice sites is delineated in Fig. 2(d). For the case, *M* = 0, BEC is self-trapped into a multi-peaked soliton by occupying several lattice sites shown in Fig. 2(c).

#### Variation of occupation number across the lattice sites

**Figure 3:**
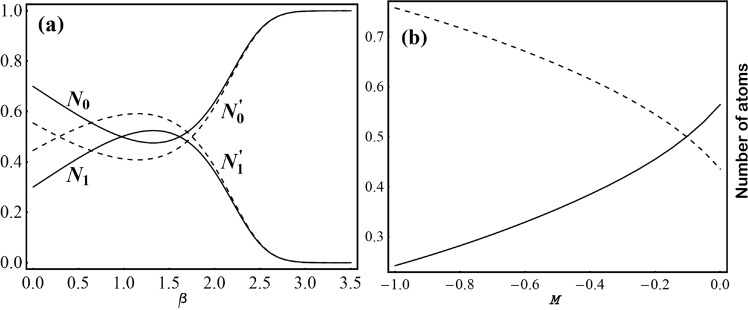
Variations of occupation number w.r.t. *β* and *M* are plotted. (**a**) Variations with *β* for *M* = 0 (dashed line) and for *M* = −0.15 (solid line). Occupation number at central site (*N*_0_, ${N}_{0}^{\text{'}}$) and rest of the lattice sites (*N*_1_, ${N}_{1}^{\text{'}}$), respectively. (**b**) Variation with *M* for *β* = 1., where occupation number at central (solid line) and rest of the lattice sites (dashed). Here, all the parameters are in dimensionless units with *γ* = 1.25, *l* = 1.84, *p* = 0, *G* = −1, *m* = 1 and *t* = 1.

To better understand the dynamics, it is worth observing the variation of condensate occupation number, which is plotted *w.r.t. β* and *M* in Fig. 3(a,b), respectively. We have taken the same physical parameter values of Fig. 2. Figure 3(a) shows the variation of condensate occupation numbers with *β* for *M* = 0 (dashed line) and *M* = −0.15 (solid line). The occupation

numbers for two different *M*’s at the central frustrated lattice site are denoted by *N*_0_ and ${N}_{0}^{\text{'}}$ and the total occupation number in all other lattice sites for two different *M*’s are denoted by *N*_1_ and ${N}_{1}^{\text{'}}$, respectively. For the case *M* = 0, atoms are getting populated in other lattice sites for 0 < *β* < 1.5, whereas for *β* > 1.5, the occupation at central site shoots up and saturates to ‘one’, beyond *β* ≈ 3.

BEC atoms flow towards the center of the trap when the depth of lattice frustration increases, which can be understood from Eq. (12). For smaller *β*, the frustrated depth is negligible leading to significant difference between the minimum energy of different lattice sites. As a result, barrier height becomes large, which hinders the BEC atoms for tunneling through the nearest neighboring sites. Thus, there is large population of atom at other lattice sites (${N}_{1}^{\text{'}}$) for *β* < 1.5. But nearly after *β* = 1.5, the frustrated depth significantly enhances, resulting into the decrease of effective barrier height allowing a rapid inter-site tunneling of BEC atoms. Finally, for high magnitude of *β*, almost the entire condensate density heaps up at the central frustrated site ${N}_{0}^{\text{'}})$. For the case of *M* = −0.15, the trap profile is depicted in Fig. 2(c). Inclusion of *M* makes the localization process more faster, as delineated by the solid line in Fig. 3(b). More negative *M* means more expulsive the trap is. It will impel the atoms to move away from the central site. However, *M* is also present in the expression of the depths of BOL (*V*_1_(*t*) and *V*_1_(*t*)). Thus, increasing *M* in negative side enhances the depth of lattice frustration, allowing more atoms to tunnel towards the central site, resulting a faster localization. Therefore, both *β* and *M* are important trap parameters through which the population of condensate atoms at various lattice sites of expulsive-BOL could be controlled. It is worth to mention that the total occupation number is normalized to one, i.e., *N*_0_ + *N*_1_ = 1 and ${N}_{0}^{\text{'}}+{N}_{1}^{\text{'}}=1$.

#### Estimation of temperature and stability of the state

To make it consistent with the formal definition of temperature, we evaluate the slope of entropy against energy of the system. Entropy (S) is expressed as: $S=-{K}_{B}{\int }_{-\infty }^{\infty }\rho (x)\mathrm{ln}\,\rho (x)dx$, where *K*_*B*_ is the Boltzmann’s constant, *ρ*(*x*) is the density of the condensate. The kinetic energy in our reduced dimensionless model can be expressed as ${E}_{K}={\int }_{-\infty }^{\infty }|\frac{\partial \psi }{\partial x}{|}^{2}dx$. We numerically evaluate the differential changes of entropy against energy for the obtained solution for two different values of *β*: 1.5 and 3.0 for *M* = 0. The calculated values are $-14.91\,J{K}_{B}^{-1}$ and $-338.98\,J{K}_{B}^{-1}$, respectively, where the negative sign implies that the temperature is negative.

It is necessary to check whether the obtained solution is sufficiently stable to confirm a negative temperature situation. We perform a numerical stability analysis by adding a random noise *R* to the wave function in Fig. 2(b), where *R* varies between 0–0.05. It is then allowed to evolve with time and the numerical simulation of the 1D-GPE has been performed using the widely used Fourier split-step method. Here, we observe the state for 1000 iterations with a properly chosen space- and time-steps: *dz* = 0.04 and *dt* = 10^−5^, respectively. The density profile retains its shape and width except some minor fluctuation, implying the analytical solution sufficiently stable. The high stability of the condensate density in expulsive-BOL trap (*i.e*., the negative temperature state) indicates that the final chemical potential is matched throughout the sample such that no global redistribution of atoms is necessary. It must be an interesting aspect to see which parameter is responsible for greater stability of the state. Here, we carry out a comparative study to find the mean deviation of the evolved solution from the initial one after 1000 iterations. The mean deviation for *β* = 1.5 and *M* = −0.15 is 1.9 percent. However, with the increase in the *β* parameter (*β* > 2, when atoms quickly tunnel to the central site) and the strength of the anti-trapping potential (*M*) the deviation reduces drastically to only 0.3 percent for *β* = 2.04 and *M* = −0.4. This is very much consistent to the physical understanding and becomes clear from our theoretical outcome.

### Atom distillation at negative temperature

**Figure 4:**
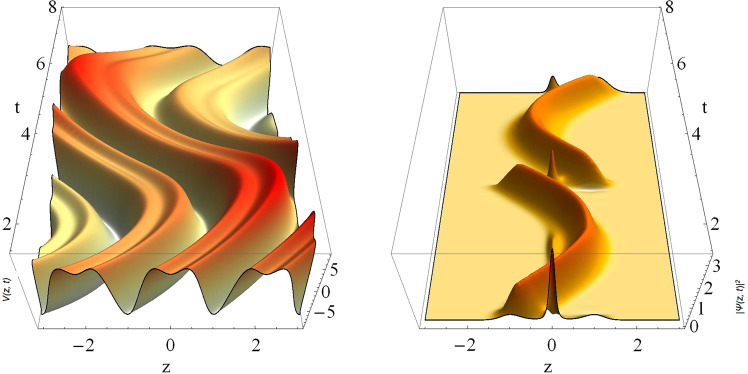
Transport of atoms from one well to another due to the asymmetry of the potential. (**a**) Potential profile, which oscillates with time, and (**b**) corresponding condensate density for parameters values, *γ* = 1.75, *β* = 2., *l* = 1.84, *M* = −0.01, *G* = −1, *m* = 1, and *p* = 2. All the parameters are made dimensionless.

**Figure 5:**
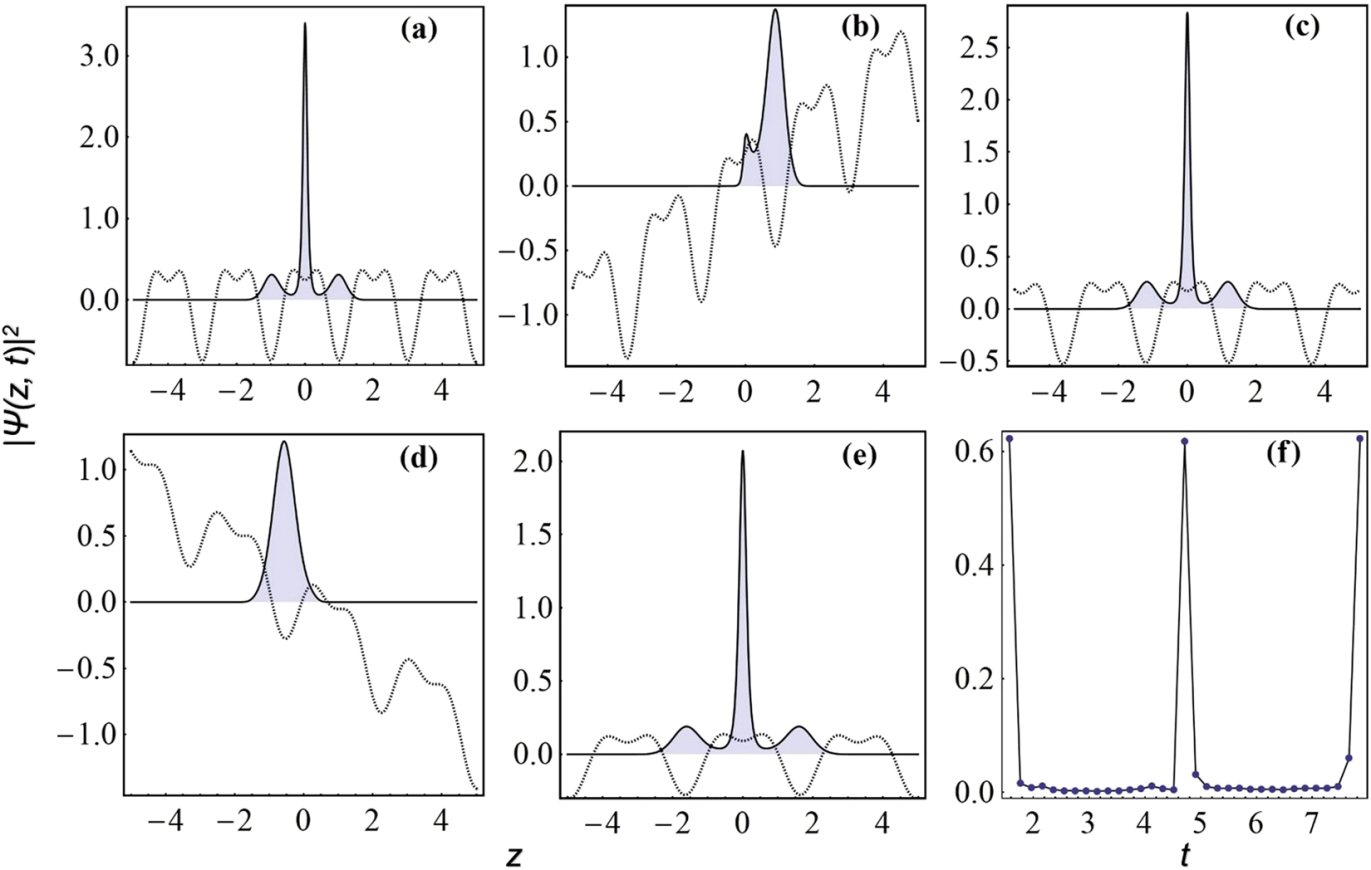
The inter-well transport of condensate atoms in an asymmetric-expulsive-BOL confinement (dashed line) and corresponding condensate densities (filled plots with solid lines) are depicted at different times: (**a**) *t* = *π*/2, (**b**) *t* = *π*, (**c)** *t* = 3*π*/2, (**d**) *t* = 2*π*, and (**e**) *t* = 5*π*/2. (**f)** Variation in occupation density at central position for *π*/2 ≤ *t* ≤ 5*π*/2. The parameter values are *γ* = 1.75, *β* = 2, *l* = 1.84, *M* = −0.01, *G* = −1, *m* = 1, and *p* = 2. All the parameters are in dimensionless units and potential is scaled by 0.1.

We demonstrate a novel mechanism for the inter-well transport of condensate atoms at negative temperature scenario^9,36^. We show that by incorporating a linear component or an asymmetry in the expulsive-BOL trap, one can induce the condensate atoms to tunnel from one well to another. In order to demonstrate this effect, we take *p* ≠ 0 in Eq. (12). Figure 4(a) depicts the effect of asymmetry on the trap configuration for the value of physical parameters: *p* = 2, *γ* = 1.75, *β* = 2., *l* = 1.84 and *M* = −0.01. Due to the presence of oscillating linear trap, Fig. 4(a) clearly exhibits the asymmetric nature and the oscillation of central lattice site with gradually decreasing depth. In order to investigate the dynamics of condensate in this confinement, we take the same parameter values of Fig. 4(a) and observe the dynamics for the whole period of oscillation (*π*/2 ≤ *t* ≤ 5*π*/2) in Fig. 4(b). The asymmetry vanishes at odd integral multiple of *t* = *π*/2 and has the periodicity *π*. Due to the presence of asymmetry in the trap, atoms are transported from one-well to another and the atomic density at the central site also varies. For providing a better interpretation of the transport of atoms in this system, we have separately depicted the 2D snapshots of external confinement and condensate density at various times: (a) *t* = *π*/2, (b) *t* = *π*, (c) *t* = 3*π*/2, (d) *t* = 2*π*, and (e) *t* = 5*π*/2 in Fig. (5), where external confinements (dashed line) and corresponding condensate densities (filled plot with solid line) are depicted, simultaneously. The present temporal evolution allows us to obtain atom-distillation at negative temperature and persisting for future experimental realization.

## Summary

In this paper, we report a large class of exact solitary wave solutions for 1D-BEC at negative temperature by exploiting a composite potential (expulsive-BOL-linear). Different combinations of external traps are explicated to broaden the scope for future applications. Condensate density is obtained for all physical scenarios at negative temperature. We have investigated the system in weak and attractive interacting domains, and obtained the bright solitary waves. The dynamics of the condensate is investigated *w.r.t*. the expulsive oscillator frequency and the intensity of the constituent lasers. The dynamics of the ultracold atoms cloud at negative temperature is in good agreement with the logical understanding and existing findings in the literature. Localization of the atoms towards the center of the trap is identified for higher magnitude of *β* and *M* in this temperature domain also. Such localization for positive temperature was reported in the literature as an analogy of Anderson localization^28^. A precise estimation of the negative temperature is provided along with a numerical stability analysis to confirm that the system under consideration belongs to negative temperature domain only. Further, utilizing the asymmetry of trap configuration, we illustrate a novel mechanism for inter-well transport of condensate atoms at negative temperature. The present method is quite generous and provides various other types of solution which can be explored further.

## Key Method Used

We analytically solve the 1D GPE of a cigar-shaped BEC in an appropriate external trap which is responsible for taking the system into the negative temperature domain. The dynamical equation which is solved can be written as

$$\begin{array}{rcl}i\frac{\partial \psi }{\partial t} & = & -\frac{1}{2}\frac{{\partial }^{2}\psi }{\partial {z}^{2}}+g(z,t)|\psi {|}^{2}\psi +[M(t){z}^{2}+{V}_{1}(t)\cos \,\mathrm{[2}l\{N(t)z+O(t)\}]\\  &  & +\,{V}_{2}(t)\cos \,[l\{N(t)z+O(t)\}]+P(t)z]\psi +i\tau (z,t)\psi \mathrm{}.\end{array}$$

Considering an appropriate ansatz solution and the travelling coordinate will result a number of consistency conditions, which upon solving give the expressions of condensate density, nonlinearity, gain or loss and phase of the system. These relations also provide the connection between the solution parameters and system parameters, allowing an efficient control over the system dynamics. The present analytical model is demonstrated with all the important steps in sequence to have clear understanding to the readers. The obtained solution is quite general as it is capable of producing cnoidal wave, dark solitary excitation etc in addition to the localized bright soliton.
